# Migration of Intrauterine Device Into the Bladder: A Case Report

**DOI:** 10.7759/cureus.72361

**Published:** 2024-10-25

**Authors:** Qing Xue, Minghan Chai, Jie Zhang, Shuting Liu, Demin Duan

**Affiliations:** 1 Gynecology, Shandong Shanxian Central Hospital, Heze, CHN

**Keywords:** bladder malposition, clinical presentation, contraceptive complications, diagnosis, intrauterine device, patient management

## Abstract

Complications associated with intrauterine contraceptive devices (IUDs), particularly those involving migration into the bladder, are infrequent occurrences yet clinically significant. The clinical presentation of patients with IUD displacement into the bladder is frequently vague and non-specific, posing a challenge for timely and accurate diagnosis. This report details the case of a patient who presented with an IUD malpositioned into the bladder, initially manifesting solely as urinary frequency. Subsequent evaluations confirmed partial displacement of the IUD into the bladder. This case underscores the paramount importance of maintaining a heightened level of suspicion in patients exhibiting suggestive symptoms, advocating for prompt and precise diagnosis even in the absence of overt or severe manifestations. Furthermore, it shares the management strategies and treatment approaches employed for such patients, aiming to offer guidance and serve as a reference for clinicians encountering cases of partial IUD displacement into the bladder.

## Introduction

In recent years, with the advancement of medical research and the continuous expansion of clinical practice, the application scope of intrauterine contraceptive devices (IUDs) has significantly surpassed their traditional contraceptive function. They are being exploratorily used in the management of menorrhagia and endometrial polyps and as an adjuvant therapy for early-stage endometrial cancer [1,2]. This trend has propelled a steady increase in the global usage rate of IUDs. As a cost-effective, long-acting, reversible, and low-failure-rate contraceptive method [3], IUDs have garnered favor among a broad spectrum of women, emerging as a vital option in contraceptive strategies.

However, despite the numerous advantages of IUDs, their associated side effects should not be overlooked. The most common include bleeding, pain, and infection. Of particular concern is uterine perforation, a serious complication with a cumulative incidence of 0.2% after one year and 0.6% after five years, posing a potential threat to women's health [4]. Inexperienced physicians, anatomical disorders of the uterus and cervix, and inappropriate timing of IUD placement are among the most significant risk factors predisposing to IUD displacement [5]. The clinical manifestations of such ectopic cases are often nonspecific, and there is currently no unified standardized treatment protocol. The management of an intraperitoneally migrated IUD is typically achieved through laparoscopic surgery, yet in certain cases, open abdominal surgery may be necessary [6]. Furthermore, a displaced IUD can also be effectively retrieved using cystoscopy.

In light of this, the present article aims to provide a detailed exposition of a rare case of IUD migration to the bladder. The intent is to offer valuable insights and guidance for obstetrician-gynecologists who encounter such complex situations. It is hoped that by sharing this case, a deeper understanding of IUD complications, especially ectopic issues, would ensue. This will optimize diagnostic and treatment pathways, enhancing patient quality of life and safety.

## Case presentation

A 39-year-old female patient presented to the urology department with urinary frequency and dyspareunia without any apparent cause for the past six months, occasionally accompanied by urinary pain, but without hematuria or post-coital bleeding. She had regular menstrual cycles, moderate flow, and no dysmenorrhea. Her last menstrual period was two weeks prior to the presentation. She had a history of two cesarean sections 20 and 18 years prior to presentation, followed by three induced abortions. Two years prior to the presentation, she had an IUD inserted for contraceptive purposes at a local hospital without follow-up.

**Figure 1:**
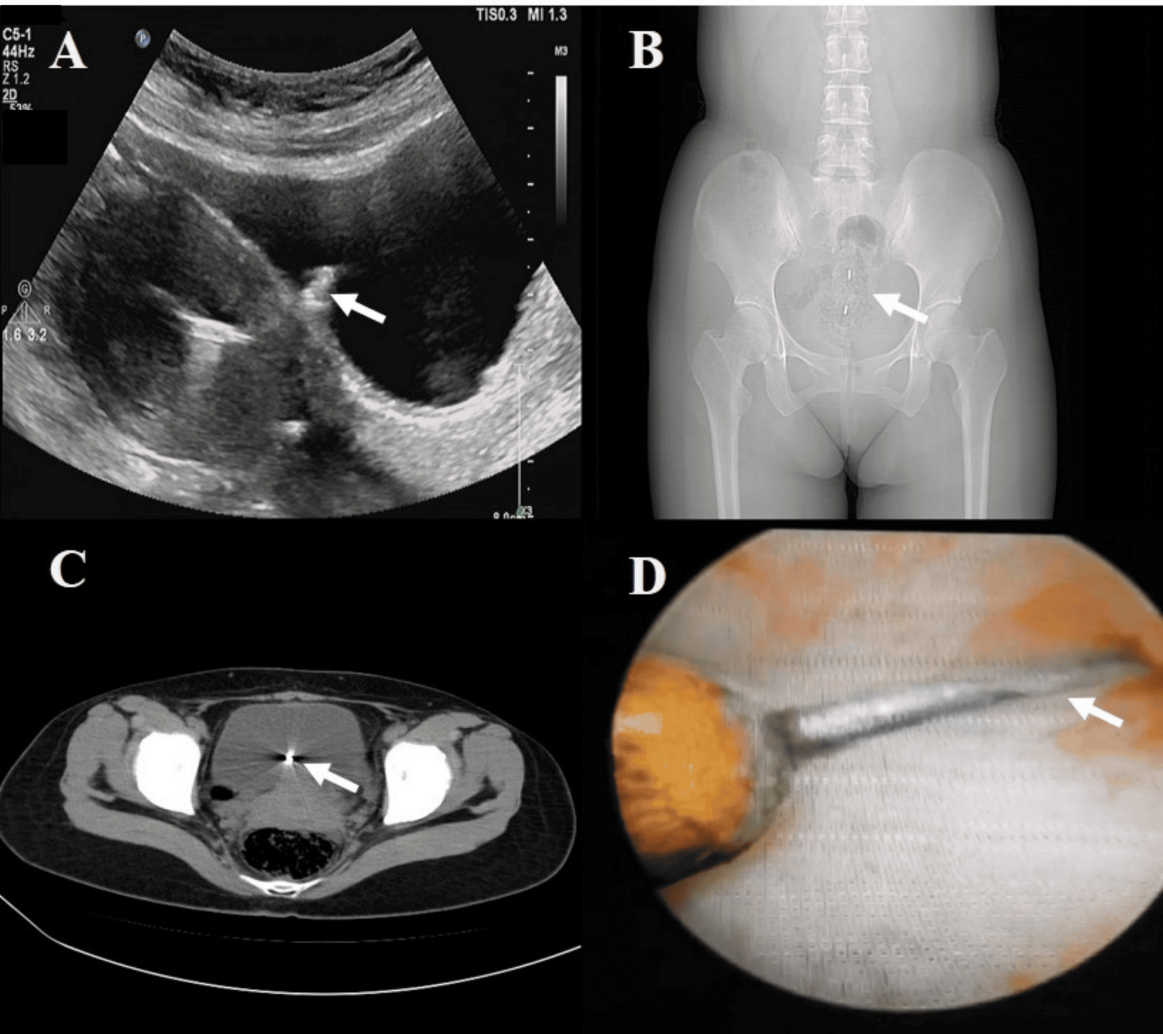
Preoperative examination: (A) Transabdominal color Doppler ultrasonography; (B) Pelvic plain film (prone position); (C) Abdominal and pelvic CT scan; (D) Cystoscopic examination.

A urine routine test at the urology department on presentation showed leukocyte esterase at 1+, and an ultrasound examination suggested a strong echo in the bladder, with a possible partial IUD embedded (Figure 1A). The urologist advised the patient to be admitted for treatment. Gynecological examination revealed normal external genitalia, a smooth vagina, a smooth cervix, an anteverted uterus with significant tenderness in the anterior fornix, and no apparent abnormalities in the adnexal regions.

Due to her work-related issues, the patient was admitted to the hospital a week later with a diagnosis of "ectopic IUD." After admission, CT scan indicated the presence of an IUD in the uterine cavity, forked in shape, with the upper branch in the uterine cavity and the lower branch penetrating the myometrium and bulging towards the posterior bladder wall, surrounded by artifacts that made it difficult to assess whether it had penetrated the bladder wall (Figure 1B, 1C). Cystoscopy revealed a foreign body about 3x1 cm in size on the posterior wall of the bladder (Figure 1D). 

**Figure 2:**
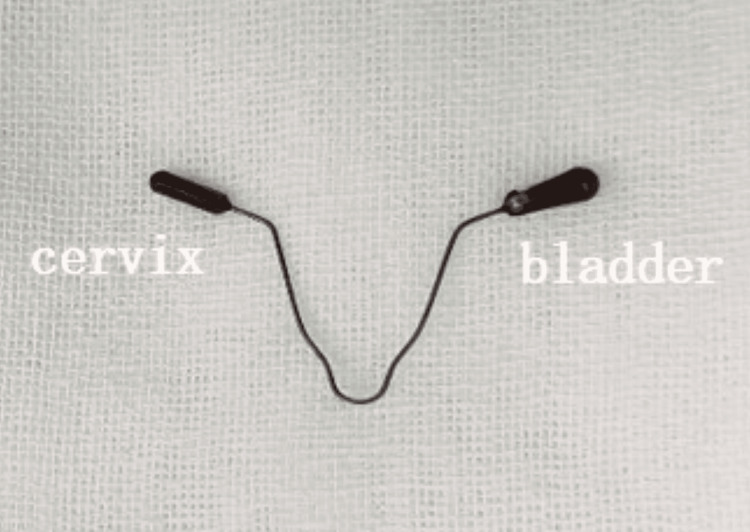
γ-shaped intrauterine contraceptive device.

After excluding surgical contraindications, hysteroscopy revealed a partial "γ"-shaped IUD in the uterine cavity (Figure 2), with one arm penetrating through the lower end of the anterior uterine wall into the myometrium. Laparoscopy showed the IUD penetrating the bladder through the attachment site of the vesicouterine peritoneal reflection at the lower segment of the anterior uterine wall. 

**Figure 3:**
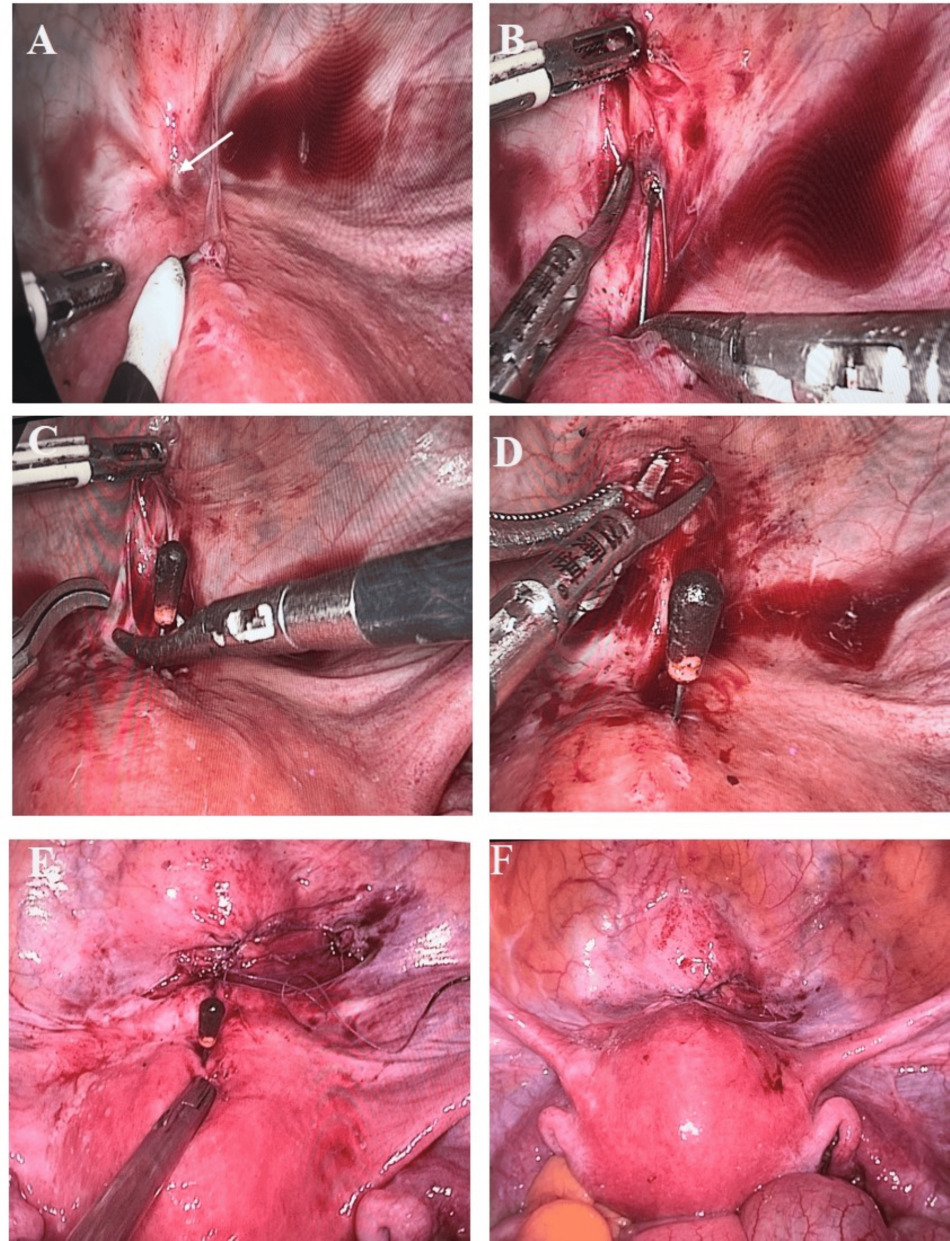
Surgical procedure: (A-C) Under laparoscopic visualization, the vesicouterine peritoneal reflection is incised, and one arm of the IUD, which is located in the bladder, is carefully extracted; (D-E) The bladder sinus tract is then excised and the edges are sutured; (F) Finally, the intrauterine contraceptive device is entirely removed. IUD: intrauterine contraceptive device

The vesicouterine peritoneal reflection was then opened to expose the metallic part of the IUD, which was gently pulled out of the bladder (Figure 3A-3C). Scissors were used to trim the tissue at the bladder sinus to create a fresh wound, the bladder wall was repaired with absorbable sutures in layers, and the vesicouterine peritoneal reflection was closed (Figure 3D, 3E). Under laparoscopic surveillance, an oocyte forceps was used to clamp the IUD through the vagina and gently pull it out intact. There was slight bleeding from the sinus tract in the anterior uterine wall, which was stopped with bipolar electrocoagulation (Figure 3F). The uterus and bilateral adnexa were explored and found to be normal, and after checking for no bleeding, the gas was evacuated, and all puncture sites were sutured. The intraoperative blood loss was 5 ml, and a urinary catheter was left in place for 14 days. After catheter removal, the patient's urine flow returned to normal.

## Discussion

The low cost and high contraceptive efficacy of IUDs make them a popular choice for many women. Approximately 14.3% of women of reproductive age worldwide use IUDs, with 64% of these users residing in China. After the insertion of an IUD, the possibility of uterine perforation and displacement is two per 1,000 [7]. Studies have identified the shape of the IUD (levonorgestrel-releasing intrauterine system), breastfeeding, first-time use, history of multiple abortions, poor healing of uterine scars after cesarean section, and inexperienced operators as high-risk factors for uterine perforation [8]. In this case, the patient's use of a "γ" type IUD, history of cesarean section, and multiple abortions may have contributed to the IUD's displacement.

Patients with IUD perforation and displacement often lack specific symptoms, and these conditions are typically identified through abdominal X-ray examinations and ultrasound imaging [9,10]. Particularly for patients with high-risk factors, regular post-insertion imaging studies can aid in the early detection of any issues, thereby facilitating timely intervention and prevention of severe complications. The patient in the current report underwent an ultrasound examination due to an increased frequency of urination, which revealed a strong echo in the bladder. Subsequent cystoscopic examination visualized one end of the IUD, indicating that an IUD displacement had occurred.

IUDs often migrate through the posterior uterine wall into the abdominal cavity, where they can become encapsulated by the omentum, and there have been reported cases of IUDs migrating to the intestines [11,12]. Cases such as the present one in which the IUD was partially displaced into the bladder, are less commonly reported [13]. For IUDs displaced to the omentum or intestines, laparoscopic surgery is commonly used for removal. For IUDs displaced into the bladder, cystoscopy is crucial for determining the surgical approach. For IUDs completely displaced into the bladder, a cystoscopic procedure can be chosen to remove the IUD [14,15]. However, in this patient, cystoscopy revealed that only one arm of the IUD was in the bladder, and hysteroscopic examination found the other arm still within the uterine cavity. Therefore, laparoscopic surgery was selected to remove the displaced IUD and to repair the bladder wall, minimizing the risk of forming a vesicouterine fistula.

## Conclusions

Although IUDs are a common and effective method of contraception, they carry certain risks. Particularly for individuals with high-risk factors, experienced practitioners, appropriate timing for insertion, and the right type of IUD can significantly reduce the occurrence of IUD displacement. Regular post-insertion imaging can promptly detect related complications such as expulsion, perforation, or displacement. Should displacement occur, imaging studies can be used to determine the location of the displacement, allowing for the selection of the appropriate treatment strategy and minimizing damage to organs.
